# Antithrombotic Therapy With Ticagrelor in Atrial Fibrillation Subjects After Percutaneous Coronary Intervention

**DOI:** 10.3389/fcvm.2021.745549

**Published:** 2021-10-12

**Authors:** Wenbin Lu, Yu Wang, Lijuan Chen, Yongjun Li, Rui Zhang, Zhongpu Chen, Jinchuan Yan, Mingming Yang, Bing Han, Zhirong Wang, Shenghu He, Lianglong Chen, Xiang Wu, Hesong Zeng, Likun Ma, Guoping Shi, Jianrong Yin, Jiyan Chen, GenShan Ma

**Affiliations:** ^1^Department of Cardiology, ZhongDa Hospital Affiliated With Southeast University, Nanjing, China; ^2^Department of Cardiology, Affiliated Hospital of Jiangsu University, Jiangsu, China; ^3^Department of Cardiology, Central Hospital of Xuzhou City, Xuzhou, China; ^4^Department of Cardiology, The Affiliated Hospital of Xuzhou Medical University, Xuzhou, China; ^5^Department of Cardiology, Northern Jiangsu People's Hospital Affiliated With Yangzhou University, Yangzhou, China; ^6^Department of Cardiology, Union Hospital Affiliated With Fujian Medical University, Fuzhou, China; ^7^Department of Cardiology, Affiliated Hospital of Nantong University, Nantong, China; ^8^Department of Cardiology, Tongji Hospital Affiliated With Tongji Medical College, Huazhong University of Science and Technology, Wuhan, China; ^9^Department of Cardiology, Anhui Provincial Hospital, Hefei, China; ^10^Department of Cardiology, Rugao People's Hospital, Nantong, China; ^11^Department of Cardiology, Pizhou People's Hospital Affiliated With Xuzhou Medical University, Xuzhou, China; ^12^Department of Cardiology, Guangdong Provincial People's Hospital, Guangdong, China

**Keywords:** antithrombic therapy, atrial fibrillation, drug eluting stent, percutaneous coronary intervention, coronary artery disease

## Abstract

**Background:** Warfarin, along with aspirin and clopidogrel, has long been recommended for patients with atrial fibrillation (AF) who are undergoing percutaneous coronary intervention with a drug-eluting stent (PCI-DES). However, this triple therapy has been known to increase the risk of bleeding complications. Meanwhile, there is no evidence from prospective trials on the use of ticagrelor in a dual therapy. We here aimed to compare the antiplatelet drug ticagrelor as a dual antithrombotic agent to aspirin and clopidogrel in bleeding events.

**Methods:** In this multicenter, active-controlled, open-label, randomized trial, patients with AF taking warfarin who had undergone PCI-DES were randomly assigned to the ticagrelor therapy group (Dual group) or the clopidogrel plus aspirin therapy group (Triple group). The primary and secondary endpoints were overall bleeding events and major bleeding events, respectively, according to the Thrombolysis in Myocardial Infarction (TIMI) criteria at 6 months. Cardiovascular events [re-PCI, surgical bypass, myocardial infarction (MI), heart failure, rehospitalization due to angina pectoris, stent thrombosis and death due to cardiovascular causes] at 6 months were also recorded.

**Results:** A total of 296 patients from 12 medical centers in China were randomized after PCI-DES to either the Dual therapy group (*n* = 148) or the Triple group (*n* = 146) for 6 months. The overall incidence of bleeding events at 6 months was 36.49% in the Dual therapy group and 35.62% in the Triple group [hazard ratio, 0.930; 95% confidence interval (CI), 0.635 to 1.361; *P* = 0.7088]. The incidence of the secondary endpoint over 6 months was 4.73% in the Dual therapy group and 1.37% in the Triple group (hazard ratio, 0.273; 95% CI, 0.057 to 1.315; *P* = 0.1056). Cardiovascular event occurrence was also comparable in both groups at 6 months (18.24 vs. 16.44%; hazard ratio, 0.845; 95% CI, 0.488 to 1.465; *P* = 0.5484).

**Conclusions:** The incidence of total bleeding events in AF patients treated with ticagrelor was comparable to that in patients treated with clopidogrel plus aspirin at 6 month; Meanwhile, the incidence of cardiovascular events were also comparable between the groups.

**Clinical Trial Registration:** MANJUSRI, ClinicalTrials.gov# NCT02206815, 2014, August 1^st^

## Introduction

Chronic treatment with oral anticoagulants (e.g., warfarin) is essential for most atrial fibrillation (AF) patients with CHA_2_DS_2_VASc scores ≥ 2. Treatment of patients with AF who have undergone percutaneous coronary intervention with a drug-eluting stent (PCI-DES) further requires follow-up antiplatelet therapy to minimize thrombotic events. Triple therapy, such as warfarin plus dual antiplatelet agents (DAPT), aspirin and clopidogrel, has been used for many years in patients with AF who have undergone PCI-DES (1).

The European Society of Cardiology recommended a short period of therapy with VKA, aspirin, and clopidogrel for such patients (2). The 2019 AHA/ACC/HRS guidelines for the management of patients with AF suggested that “Triple therapy should be administered to these patients with bare-metal stents for 1–3 months and much longer in patients with a drug-eluting stent (3–6 months) followed by one anticoagulant plus clopidogrel 75 mg/day or aspirin 100 mg/day” (3).

RCT studies as well as retrospective analyses all suggested that the combination of oral anticoagulation with a P2Y12 inhibitor and aspirin in patients with AF undergoing PCI-DES is associated with a high bleeding risk (4, 5). Triple antithrombotic therapy, particularly if consisting of a OAC, aspirin and a P2Y12 inhibitor, is associated with a increasing of bleeding, including major and intracranial hemorrhages (6). However, pooled data from meta-analysis (7) have shown a possible increase of ischemic events in the dual therapy with clopidogrel, for example Galli et al. confirmed that dual therapy with clopidogrel with a significant increase of stent thrombosis risk in the overall population and a significant 43% increase of MI in the ACS/PCI subgroup (8) sparkling the interest for the use of alternative antithrombotic agents. In addition to this, clopidogrel, but not ticagrelor, is characterized by an interindividual variability in the pharmacodynamic profile, leading to insufficient platelet inhibition and increased ischemic events in up to 40% of treated patients and thus Most recent investigations support the clinical benefit of a genetic guided selection of antiplatelet therapy in patients undergo PCI (i.e., switching to prasugrel or ticagrelor) (9, 10).

Recently, randomized clinical trials and recent evidence have supported the ESC recommendation for dual antithrombotic therapy in patients with AF who have undergone PCI-DES (6, 11). As is well-known, the recent guidelines (2018–2020 ECS Guidelines) supported the current trend of antithrombotic therapy using a combination of one antiplatelet drug and one anticoagulant (12–14), for example,2020 ESC Guidelines presented that In AF patients with ACS undergoing an uncomplicated PCI, early cessation (<1 week) of aspirin and continuation of dual therapy with an OAC and a P2Y12 inhibitor for up to 12 months is recommended if the risk of stent thrombosis is low or if concerns about bleeding risk prevail over concerns about risk of stent thrombosis, irrespective of the type of stent used. However,1 week triple therapy with clopidogrel can be associated to increased ischemic events.

The TWILIGHT trial proved that ticagrelor monotherapy was associated with a lower incidence of clinically relevant bleeding than ticagrelor plus aspirin, without higher risks of death, myocardial infarction, or stroke, again suggesting that ticagrelor is a more potent antiplatelet drug (15, 16).

The recent AUGUSTUS study was designed for AF patients who had ACS or had undergone PCI and were taking a P_2_Y_12_ inhibitor to receive apixaban or warfarin and aspirin or a matching placebo for 6 months (17, 18). However, more than 90% of the individuals in this trial were treated with clopidogrel instead of more potent agents, such as ticagrelor. Among published clinical studies such as PIONEER trial, ENTRUST trial and Re-Dual trial, the Re-Dual study included the most patients received ticagrelor were only 12% (19–21). The use of ticagrelor can potentially overcome the increased rate of ischemic events as the rate of non-responders to this drug is very low.

On the other hand, triple therapy with ticagrelor in these previous trials was somewhat unfounded and in particular lacked the support of ESC guidelines. Thus, the rationale for our study is that ticagrelor were used only in a minority of the 4 RCTs on the topic (PIONEER, RE-DUAL, AUGUSTUS and ENTRUST-AF), which implied that there are not many data from prospective studies on a dual therapy including this potent P2Y12 inhibitors.

Therefore, we performed the present study to compare the combination of ticagrelor and warfarin with traditional triple therapy, with the aim to evaluate the safety and efficacy of ticagrelor as one of dual antithrombins.

## Methods

### Study Design

The trial rationale and design were published previously (22). In brief, the MANJUSRI trial was a prospective, multicenter, open-label, randomized clinical trial that compared the potent antiplatelet drug ticagrelor with the traditional antithrombotic drugs clopidogrel plus aspirin in patients with AF taking warfarin and receiving PCI-DES (all of the patients in the trial received drug-eluting stents). This clinical trial was registered with ClinicalTrials.gov (NCT02206815). The study was performed in accordance with the provisions of the Declaration of Helsinki and the International Conference on Harmonization Good Clinical Practice guidelines. The institutional ethics committee associated with the participating centers approved the trial protocol. All of the patients provided written informed consent.

### Study Population

Eligible patients were recruited from 12 participating centers in China from September 2014 to February 2019. The inclusion criteria were men or nonpregnant women ≥ 18 and ≤ 75 years of age with a severe coronary lesion (≥ 75% angiographically or fractional flow reserve, FFR <0.8) with an indication for coronary stent implantation and patients with a CHA_2_DS_2_-VASc score ≥ 2. The exclusion criteria are listed in Supplementary Table 1. CHA_2_DS_2_-VASc scores, which reflect the risk of stroke, and HAS-BLED scores, which reflect the risk of bleeding, were assessed in all of the patients.

### Randomization and Trial Regimen

Patients who met all of the inclusion criteria and none of the exclusion criteria were randomly assigned at a 1:1 ratio to receive ticagrelor and warfarin (Dual therapy group) or clopidogrel + aspirin + warfarin (Triple group) just after PCI-DES. The randomization sequence and allocation were accomplished using sealed envelopes containing a computer-generated sequence (generated at ZhongDa Hospital Affiliated with Southeast University). The randomized enrollment progress and patient distribution are shown in Supplementary Figure 1. All of the patients in the trial took warfarin, and the doses were adjusted to reach a target international normalized ratio (INR) within the range of 2.0 to 2.5. Before PCI-DES, a loading dose of 180 mg of ticagrelor or 300 mg of clopidogrel and 300 mg of aspirin were administered. After PCI-DES, patients on Triple group received clopidogrel 75 mg/day + aspirin 100 mg/day, whereas patients in the Dual therapy group received ticagrelor 90 mg/bid. The use of proton pump inhibitors to reduce the risk of bleeding was recommended but not mandatory. Follow-ups were performed during routinely scheduled outpatient visits at 1, 3, and 6 months and via telephone calls at 15 days and 7 months after randomization. After 6 months, the patients were transitioned from their 2-trial interventions to receive antiplatelet and anticoagulant therapy at the discretion of the attending physician according to the local standard of care.

### Outcomes and Endpoints

The primary endpoint of the study was the occurrence of overall bleeding events (minimal, minor, and major) during the first 6 months of follow-up. The secondary endpoint was the occurrence of major bleeding events during the 6-month follow-up. Bleeding events in the trial were assessed according to the Thrombolysis in Myocardial Infarction (TIMI) bleeding criteria (23–26): (1) Major: any intracranial bleeding; clinically overt signs of hemorrhage associated with a drop in hemoglobin of ≥5-g/dL or a ≥15% absolute decrease in hematocrit; and fatal bleeding; (2) Minor: clinically overt signs (including imaging), resulting in a hemoglobin drop of 3- to <5-g/dL or a ≥10% decrease in hematocrit; no observed blood loss: ≥4 g/dL decrease in the hemoglobin concentration or ≥12% decrease in hematocrit; requiring medical attention: any overt sign of hemorrhage that met one of the following criteria and did not meet the criteria for a major or minor bleeding event, as defined above; requiring intervention: medical treatment to stop or treat bleeding, including changing the dose of the study drug; leading to or prolonging hospitalization; and prompting evaluation (unscheduled visit to a healthcare professional and diagnostic testing); (3) Minimal: any overt bleeding event not meeting the criteria above; any clinically overt sign of hemorrhage (including imaging) associated with a <3-g/dL decrease in hemoglobin concentration or <9% decrease in hematocrit.

### Assessment of Cardiovascular Events

Cardiovascular events reported were re-PCI (percutaneous coronary intervention) or surgical bypass, MI (myocardial infarction), heart failure and rehospitalization due to angina pectoris, stent thrombosis and death due to cardiovascular causes, all of which were in accordance with the Academic Research Consortium criteria. Standardized questions were used to assess cardiovascular events, and the use of medications. A committee of clinical events that was unaware of treatment allocations adjudicated and verified all events that required medical attention from the medical records of the referring doctors and hospitals.

### Statistical Analysis

Data management and statistical analyses were performed using SAS software, version 9.4 (SAS Institute, USA).

Based on previous research mainly retrospective analysis at the time, we anticipated a bleeding complication rate of 18% in the dual ticagrelor therapy group and 30% in the clopidogrel plus aspirin group at 6 months. The proportion of patients dropping out of the treatment and the control groups was assumed to be 5%. The sample size was estimated at 296 subjects.

The primary and secondary endpoints were the first occurrences of events. The primary analysis compared the time from randomization to the first occurrence of any event in the composite endpoint using the log-rank test.

The null and alternative hypotheses were the following.

H0: The distribution of the first occurrence of bleeding in the 2 groups is the same.
H1: The distribution of the first occurrence of bleeding in the 2 groups is not the same.

The hypotheses were tested at an overall significance level of 5% (2 sided). Patients who failed to record any event at the primary composite efficacy endpoint were censored at the close of the study (i.e., date of the end-of-treatment visit) or at the time of the last available information, if not earlier. Kaplan-Meier and Cox proportional hazard regression estimates of the cumulative risk for the first occurrence of any event were calculated in the 2 groups. The hazard ratio and 95% confidence intervals (CIs) are reported.

### Role of the Funding Source

The sponsors of the study played no role in the entire process, including the study design, data collection, and data analysis. The corresponding author is fully responsible for the trial and manuscript publishing.

## Results

### Baseline Characteristics of Patients With AF

**Figure 1:**
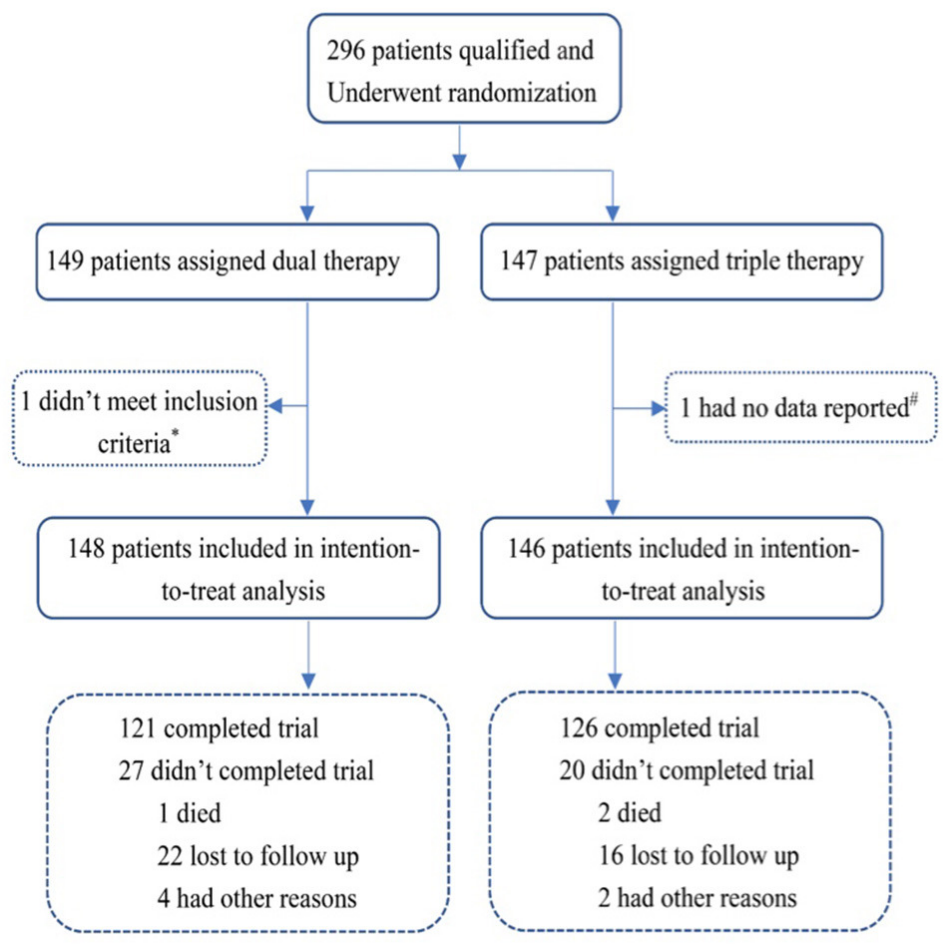
Randomization and follow-up. *One patient in the ticagrelor therapy group did not meet the inclusion criteria immediately after randomization; #one patient in the Triple therapy group had no data reported. Dual therapy: ticagrelor therapy group; Triple therapy: clopidogrel plus aspirin group.

From September 2014 to February 2019, a total of 296 patients from 12 sites in China were enrolled. Totals of 148 and 146 AF patients were assigned to the ticagrelor therapy group (Dual group) and clopidogrel plus aspirin (Triple group) therapy group, respectively (Figure 1). After a follow-up period of 6 months for both groups, the final collection of follow-up data occurred on February 20, 2019. A total of 3 patients died during the study period. The direct cause of death of a patient in the ticagrelor therapy group was heart failure; One patient died of trauma following a fall in Triple therapy group, and the other also in the Triple group died of acute exacerbation of renal insufficiency.

The mean age of the patients in the trial was 69.39 years old. In all, 65.54% of patients in the Dual therapy group and 62.33% in the Triple therapy group were male. Clinical comorbidities, including hypertension, diabetes, and history of stroke/TIA, and the use of medication were comparable between the 2 groups (Table 1). The average CHA_2_DS_2_-VASc score was 3.41 in the ticagrelor therapy group and 3.22 in the Triple therapy group. The mean HAS-BLED scores were 2.01 in the ticagrelor therapy group and 1.97 in the triple therapy group. The mean INR at randomization was 2.08 in the ticagrelor therapy group and 2.13 in the Triple therapy group, respectively. Baseline procedural characteristics were similar between the two groups, including PCI-related vessels and periprocedural treatments, are shown in Table 2.

**Table 1:** Characteristics of the patients at baseline.

**Characteristics**	**Ticagrelor/dual group (*n* = 148)**	**Triple group (*n* = 146)**	***P*-value**
Age (years, mean ± SD)	69.33 ± 8.25	69.45 ± 7.65	0.8909
Sex (male), N	97 (65.54%)	91 (62.33%)	0.6274
BMI (kg/m^2^, mean ± SD)	25.53 ± 2.99	25.85 ± 4.33	0.4608
Smoke, *N*	41 (27.70%)	27 (18.49%)	0.0611
**Comorbidities**		
Diabetes	52 (35.14%)	41 (28.08%)	0.1935
Hypertension	111 (75.00%)	105 (71.92%)	0.5981
Hypercholesterolemia	4 (2.70%)	5 (3.42%)	0.7488
History of stroke/TIA	40 (27.03%)	30 (20.55%)	0.1922
NYHA(III-IV)	76 (51.35%)	68 (46.58%)	0.4127
Peripheral vascular diseases	4 (2.70%)	7 (4.79%)	0.3447
**Medication**		
β-blocker	122 (82.43%)	113 (77.40%)	0.3099
Statin	132 (89.19%)	138 (94.52%)	0.1348
ACEI/ARB	93 (62.84%)	88 (60.27%)	0.7193
PPI	58 (39.19%)	61 (41.78%)	0.7216
Nitrates	70 (47.30%)	65 (44.52%)	0.6417
INR at randomization	2.08 ± 0.84	2.13 ± 0.60	0.5698
CHA_2_DS_2_-VASc score	3.41 ± 1.56	3.22 ± 1.48	0.4364
HASBLED score	2.01 ± 1.09	1.97 ± 1.14	0.7104

*Data are expressed as the mean ± standard deviation and n (%). ACEI, angiotensin-converting enzyme inhibitor; ARB, angiotensin receptor blocker; BMI, body mass index; INR, International Normalized Ratio; NYHA, New York Heart Association classification grading of cardiac function; PPI, proton pump inhibitor; TIA, transient ischemic attack*.

**Table 2:** Baseline Procedural Characteristics.

**Characteristics**	**Dual therapy group (*n* = 148)**	**Triple group (*n* = 146)**
ACS patients	36 (24.33%)	28 (19.18%)
Radial artery	145 (97.97%)	142 (97.26%)
**Number of drug-eluting stents**		
1	106 (71.62%)	98 (67.12%)
2	34 (22.97%)	37 (25.34%)
≥3	8 (5.41%)	11 (7.53%)
**PCI vessel**		
LM	1 (0.61%)	9 (5.33%)
LAD	95 (57.93%)	90 (53.25%)
LCX	34 (20.73%)	34 (20.12%)
RCA	34 (20.73%)	36 (21.30%)
**Periprocedural treatment**		
Continuation of OAC	112 (29.79%)	111 (31.09%)
Unfractionated heparin	140 (37.23%)	129 (36.13%)
Bridging with LMWH	72 (19.15%)	79 (22.12%)
GPIIbIIIa	52 (13.83%)	38 (10.64%)

*Data are expressed as n (%). ACS, acute coronary syndrome; GPIIbIIIIa, glycoprotein IIbIIIa receptor blocker; LAD, left anterior descending artery; LCX, left circumflex artery; LM, left main coronary artery; LMWH, low-molecular-weight heparin; OAC, oral anticoagulation; PCI, percutaneous coronary intervention; RCA, right coronary artery*.

### Bleeding Events

**Figure 2:**
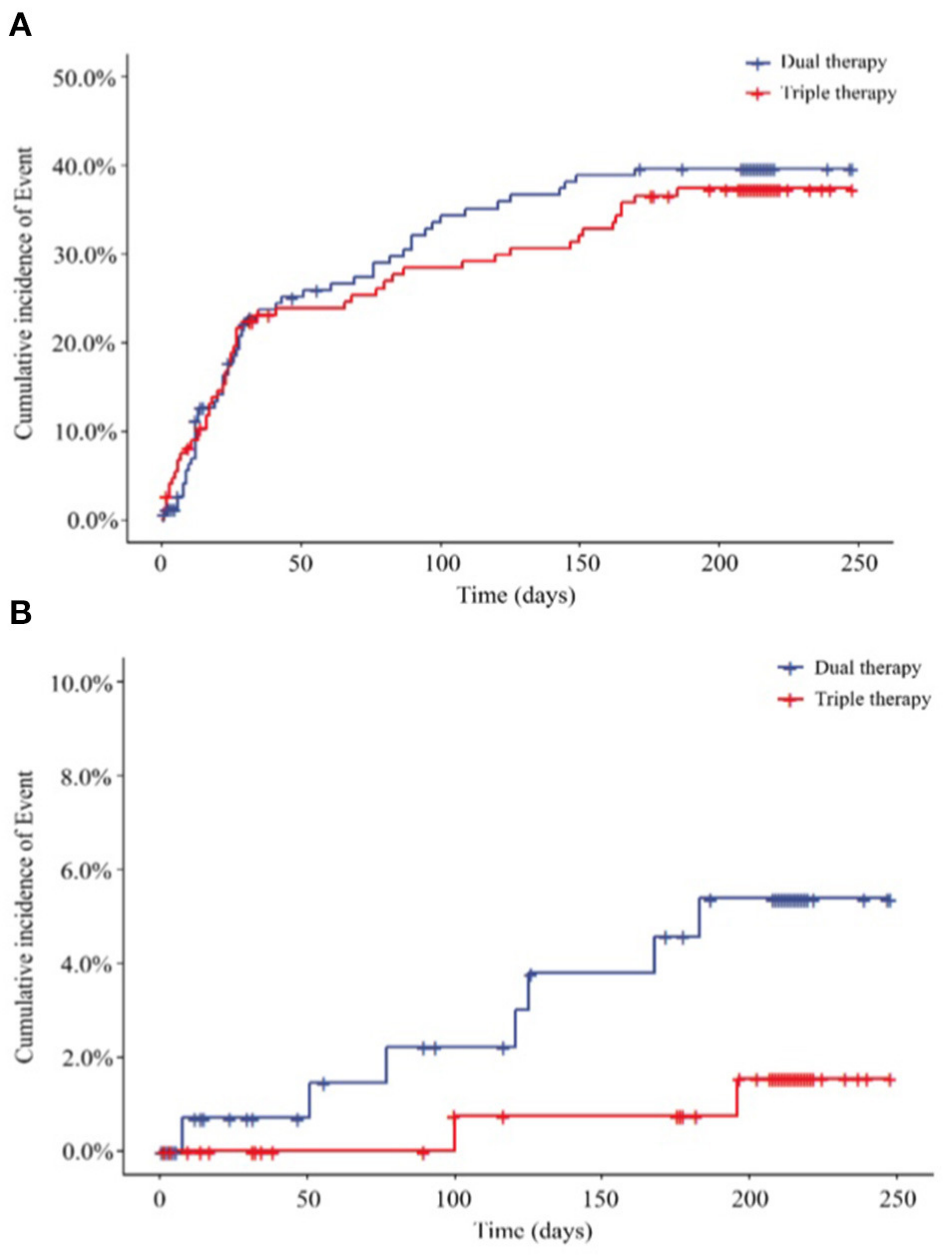
Endpoints, **(A)** Primary outcome for the incidence of total bleeding (HR 0.930; 95% CI, 0.635 to 1.361; *P* = 0.7088); **(B)** secondary outcome for the incidence of major bleeding (HR, 0.273; 95% CI, 0.057 to 1.315; *P* = 0.1056) HR, hazard ratio. Dual therapy: ticagrelor therapy group; triple therapy: clopidogrel plus aspirin group.

After a 6-month follow-up, 54 patients (36.49%) in the ticagrelor therapy group and 52 patients (35.62%) in the Triple group experienced bleeding events. The time-to-first bleeding event analysis revealed comparable bleeding rates between the 2 groups [hazard ratio (HR), 0.930; 95% CI, 0.635 to 1.361; *P* = 0.7088] (Figure 2A). Similarly, 7 (4.73%) patients in the ticagrelor therapy group and 2 (1.37%) patients in the Triple group experienced major bleeding events (HR, 0.273; 95% CI, 0.057 to 1.315; *P* = 0.1056) according to the TIMI bleeding classifications (Figure 2B).

The majority of bleeding events in the trial were minor bleeding events mainly concentrated in the nose (27.78 vs. 21.15%) and mouth (29.63 vs. 32.69%) in the ticagrelor therapy and triple therapy groups (Table 3). We observed comparable distributions and proportions of bleeding events between the 2 groups. The occurrence of multiple bleeding events especially twice bleeding seems more frequently in the Triple therapy group (36.54%) and it was 20.37% in the ticagrelor therapy group, but showed no significant difference between the groups.

**Table 3:** Bleeding Times and Specific Location of Bleeding in 2 Groups of Patients; “Others” were cases in which the bleeding site could not be identified, but were identified as bleeding by the research team due to Hb decrease.

**Bleeding specific**	**Dual therapy group (*n* = 148) primary events (*n* = 54)**	**Triple therapy group (*n* = 146) Primary events (*n* = 52)**
**Bleeding times**		
1 time	37 (68.52%)	30 (57.69%)
2 times	11 (20.37%)	19 (36.54%)
≥3 times	6 (11.11%)	3 (5.77%)
**Bleeding location**		
Intracranial	1 (1.85%)	0 (0%)
Eye	1 (1.85%)	2 (3.85%)
Mouth	16 (29.63%)	17 (32.69%)
Gastrointestinal	4 (7.41%)	2 (3.85%)
Nose	15 (27.78%)	11 (21.15%)
Respiratory tract	3 (5.56%)	2 (3.85%)
Urinary system	0 (0%)	2 (3.85%)
Skin hematoma	9 (16.67%)	9 (15.38%)
Other	5 (9.26%)	8 (15.38%)

*Data are expressed as n (%)*.

**Figure 3:**
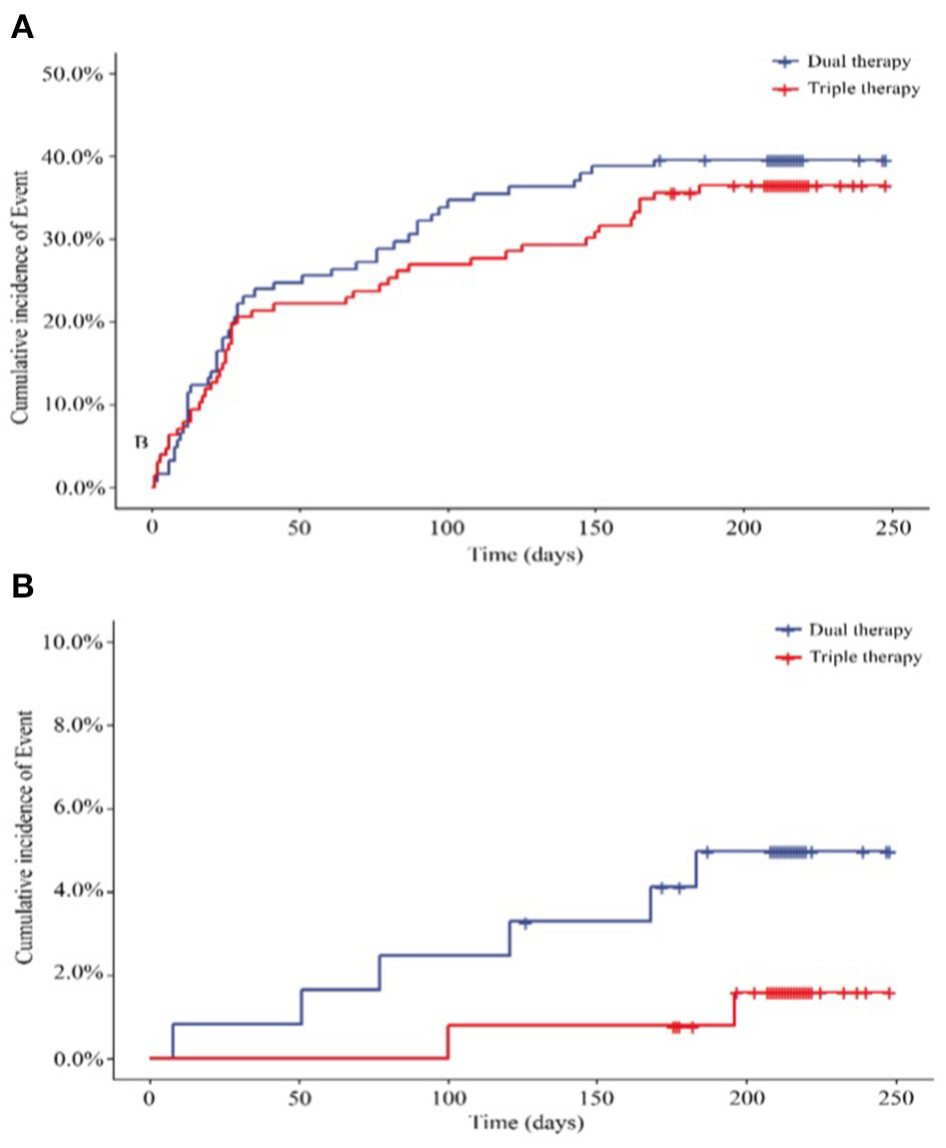
**(A)** Primary outcome for the incidence of total bleeding in PPS (*N* = 247) (HR, 0.889; 95% CI, 0.593 to 1.332; P = 0.5684); **(B)** Secondary outcome for the incidence of major bleeding (HR, 0.313; 95% CI, 0.063 to 1.552; *P* = 0.1550). HR, hazard ratio. Dual therapy: ticagrelor therapy group; triple therapy: clopidogrel plus aspirin group.

According to the data analysis and patients lost to follow-up or other reasons, we obtained 247 patients for the per protocol set (PPS). In the set, 48 of the 121 patients (39.67%) in the ticagrelor therapy group experienced total bleeding events compared with 46 of the 126 (36.51%) patients in the Triple group, representing comparable bleeding rates between the 2 groups (HR, 0.889; 95% CI, 0.593 to 1.332; *P* = 0.5684) (Figure 3A; Supplementary Table 2). Considering the secondary endpoints in the PPS, 6 (4.96%) patients in the ticagrelor therapy group experienced major bleeding events, whereas 2 (1.59%) patients in the Triple therapy group experienced major bleeding events. Time-to-event analysis showed no significant difference (HR, 0.313; 95% CI, 0.063 to 1.552; *P* = 0.1550) (Figure 3A; Supplementary Table 2) between the 2 groups.

### Ischemic Events

**Figure 4:**
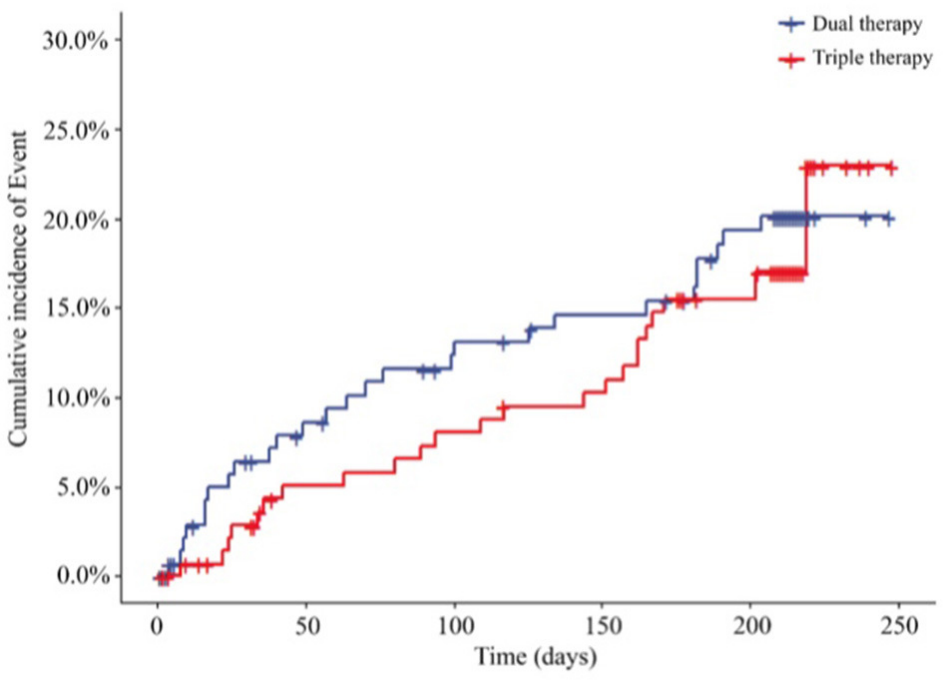
Incidence of cardiovascular event outcomes of the 2 groups (HR, 0.845; 95% CI, 0.488 to 1.465; *P* = 0.5484). HR, hazard ratio. Dual therapy: ticagrelor therapy group; triple therapy: clopidogrel plus aspirin group.

Cardiovascular events, including re-PCI, surgical bypass, MI, heart failure, rehospitalization due to angina pectoris, stent thrombosis and death due to cardiovascular causes, at the end of the 6-month follow-up are shown in Figure 4; Table 4. We reported cardiovascular events in 27 (18.24%) patients in the ticagrelor therapy group, whereas 24 (16.44%) patients in the Triple group experienced these events (HR, 0.845; 95% CI, 0.488 to 1.465; *P* = 0.5484).

**Table 4:** Specific description of cardiovascular events in 2 groups of patients.

**Cardiovascular events**	**Dual therapy group (*n* = 148) Cardiovascular events (*n* = 27)**	**Triple therapy group (*n* = 146) Cardiovascular events (*n* = 24)**
Re-PCI	3 (11.11%)	2 (8.33%)
Surgical bypass	0 (0.0%)	0 (0.0%)
Myocardial infarction	3 (11.11%)	2 (8.33%)
Heart failure	3 (11.11%)	4 (16.67%)
Rehospitalization due to angina pectoris	15 (55.55%)	15 (62.55%)
Stent thrombosis	0 (0.0%)	0 (0.0%)
Death due to cardiovascular causes	1 (3.70%)	1 (4.67%)
Others	2 (7.40%)	2 (8.33%)

“*Others” referred patients who were reported symptoms of chest tightness or chest pain or heart failure but refused to go to the hospital for further determination and were considered by the study team to be compatible with cardiovascular events. Data are expressed as n (%). PCI, percutaneous coronary intervention*.

**Figure 5:**
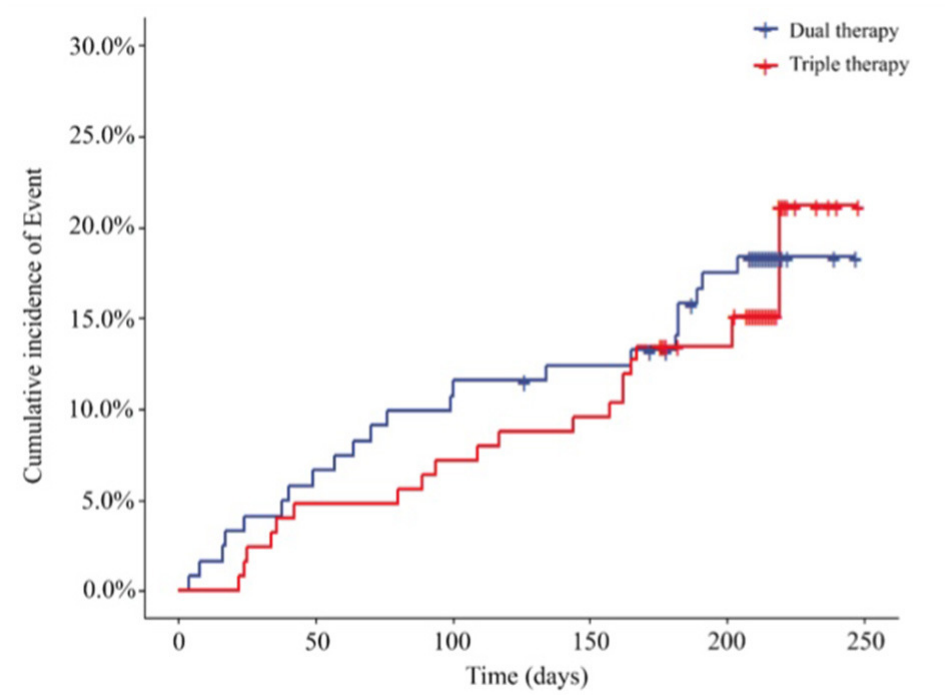
Incidence of cardiovascular event outcomes of the two groups (PPS, *N* = 247) (HR, 0.844; 95% CI, 0.461 to 1.547; *P* = 0.5836). HR, hazard ratio. Dual therapy: ticagrelor therapy group; triple therapy: clopidogrel plus aspirin group.

Furthermore, at the end of the 6-month follow-up in the PPS, 22 (18.18%) patients in the ticagrelor therapy group experienced cardiovascular events compared to 20 events (15.87%) in the Triple group. Time-to-event analysis showed no significant difference (HR, 0.844; 95% CI, 0.461 to 1.547; *P* = 0.5836) (Figure 5; Supplementary Table 3) between the 2 groups.

## Discussion

There are many issues surrounding antithrombotic treatment of patients with AF undergoing PCI that remain unresolved. Whether ticagrelor as a part of dual therapy would exhibit favorable safety and efficacy is unknown. Some meta-analysis suggested that the use of ticagrelor as part of dual or triple antithrombotic therapy is associated with significantly higher rates of clinically relevant hemorrhagic complications compared with clopidogrel (27).

What's different is that our trial primarily confirmed the safety of ticagrelor as a dual therapy regimen didn't increasing bleeding rates, especially total bleeding rates. We further found that most of the total TIMI bleeding events were minimal and minor events. This result indicates that ticagrelor, even with its potent antithrombotic effect, did not increase the risk of fatal bleeding after 6 months as dual therapy when combined with warfarin and administered to AF patients after they underwent PCI-DES.

2019 AHA/ACC/HRS guidelines suggested that in patients with AF at increased risk of stroke (based on CHA2 DS2 -VASc risk score of 2 or greater) who have undergone PCI with stenting for ACS, double therapy with a P2Y12 inhibitor (clopidogrel or ticagrelor) and dose-adjusted vitamin K antagonist is reasonable to reduce the risk of bleeding as compared with triple therapy (3). Our results provide further evidence support and supplement for the clinical application of dual therapy with different P2Y12 inhibitor. On the other hand our results are consistent with and support the trends of using dual drug regimen in patients with atrial fibrillation and PCI-DES, as recommended by recent guidelines (12, 28, 29). In the “North American Perspective 2016–2021 Update” regarding antithrombotic treatment for these patients, triple therapy was only recommended during the peri-PCI period and dual therapy as soon as possible after hospital discharge (30–32). In other words, our study suggests a possibility that a slightly stronger dual antithrombotic with ticagrelor therapy might be used immediately after PCI in AF patients, instead of 1 week to 1 month of triple antithrombotic therapy.

The MANJUSRI trial showed that, among AF patients who had undergone PCI-DES, the anticoagulation regimen warfarin plus ticagrelor resulted in comparable total bleeding events compared with Triple therapy. The difference in risk between the ticagrelor therapy group and the Triple therapy group was 6.8% (0.87 percentage points) over ~6 months of treatment. Compared to previously published trials, bleeding rates (33, 34) in our trial appeared to be amplified. This might be attributed to several factors, firstly, patients in triple group received a relatively longer time of Antithrombotic therapy, then nearly one-third of the patients in our study used glycoprotein receptor antagonists during the perioperative period and Glycoprotein IIbIIIa receptor antagonists seemed to be used more frequently in the trial of ticagrelor group.

The rates of major TIMI bleeding were higher in the ticagrelor therapy group than in the Triple group, but the difference was not statistically significant. The difference in risk between the groups was 27.5% (3.36 percentage points), however in our trial, we must consider the particularity of certain cases themselves. In the ticagrelor therapy group, only 1 of 7 major bleeding patients suffered from intracranial bleeding, and the others all experienced gastrointestinal bleeding. However, it is important to note that 2 of the patients who experienced major bleeding were at high risk at the time of enrollment. One patient had a HASBLED score ≥3, and the other patient had an INR of 4.43. Another 3 patients' specific situations were similar to those of the 2 patients in the Triple group. In conclusion, these findings suggest a relatively balanced risk of bleeding in the prevention of thromboembolism, at least to some extent, providing clinicians with one more regimen option when considering a AF patient's risk of bleeding and the risk of thromboembolic events after PCI-DES.

The strategies for dual therapy with ticagrelor that we tested incorporated two changes relative to the previous trials of antithrombotic therapy. The first change is the incorporation of ticagrelor as part of the dual therapy regimen for all patients with AF just after PCI-DES. The proportion of patients taking ticagrelor in our trial (every patient in the dual therapy group took oral ticagrelor, so the usage rate was 100%) was greater to that in the few other trials that have investigated ticagrelor as part of the regimens in patients with atrial fibrillation and PCI-DES (3–5.5% in the PIONEER trial; 7–8% in ENTRUST trial) (15, 16). The second change refers to the use of warfarin as the basic anticoagulant for the two groups of patients, instead of NOVC. This change was mainly based on economic reasons given that most Chinese patients prefer warfarin due to its relatively lower price, and at the time of enrollment, more than 90% of the patients were taking warfarin once daily as antithrombotic therapy for AF (35, 36); Secondly, access to NOACs in China was possible only after 2016.

There several limitations in the trial though we are the first trial that systematically evaluated the safety of ticagrelor as part of a dual antithrombotic regimen. First, the primary analyses showed that the safety of ticagrelor was similar to that of Triple therapy, and the result just reached a point of non-inferiority, rather than superiority, as anticipated. This outcome might be related to the potent antiplatelet effect of ticagrelor as discussed (37), and longer duration of antithrombotic administration as well as more additional antithrombotic administration during perioperative period. Secondly, We did not compare the risk of bleeding between the two groups at 1 week and 1 month, because current guidelines only recommend the shorter-term triple antithrombotic therapy (6, 28, 29, 38). Thirdly, the rates of primary endpoints of bleeding in this study were higher than those in the previous trials on this topic; in addition to over verified minor bleeding as discussed above, we suspected that to some extent this outcome might be related to increased variability in INR during the follow-up process and Asians being prone to bleeding when treated with warfarin (39, 40). In the end, the number of patients in our trial was relatively small for various reasons, including research funding, thus restricting the power of the study to detect worthwhile differences between the two groups. In addition, the progress of the trial was relatively slow due to physicians' and patients' concerns about various issues, such as safety in subcenters during the first year. However, though the trial did take us almost 5 years to complete, the patients were not highly selected, all of the data in the trial met the random requirements and the inclusion and exclusion criteria, and the two groups of patients were relatively homogenous.

## Conclusion

Patients with AF undergoing PCI-DES treated with ticagrelor and warfarin in this trial experienced an incidence of total bleeding events that was comparable to those receiving a traditional triple antithrombotic regimen consisting of clopidogrel, aspirin, and warfarin. In this study we found that a dual antithrombotic therapy with ticagrelor is associated with similar incidence of bleeding and ischemic events as compared to a triple therapy with clopidogrel among AF patients undergoing PCI, however, we did not demonstrated dual therapy using ticagrelor reduced bleeding events compared to triple therapy. Further studies are warranted to shed light on the potential benefit of implementing a dual antithrombotic therapy with potent P2Y12 inhibitor in this setting.

## Data Availability Statement

The original contributions presented in the study are included in the article/Supplementary Material, further inquiries can be directed to the corresponding author/s.

## Ethics Statement

The studies involving human participants were reviewed and approved by ICE for Clinical research of Zhongda Hospital Affiliated to Southeast University. The patients/participants provided their written informed consent to participate in this study. Written informed consent was obtained from the individual(s) for the publication of any potentially identifiable images or data included in this article.

## Author Contributions

All authors listed have made a substantial, direct and intellectual contribution to the work, and approved it for publication.

## Funding

The study was supported by AstraZeneca Pharmaceutical Co., Ltd. (No. ISSBRIL0256).

## Conflict of Interest

This study received funding from AstraZeneca Pharmaceutical Co., Ltd. The funder was not involved in the study design, collection, analysis, interpretation of data, the writing of this article or the decision to submit it for publication. The remaining authors declare that the research was conducted in the absence of any commercial or financial relationships that could be construed as a potential conflict of interest.

## Publisher's Note

All claims expressed in this article are solely those of the authors and do not necessarily represent those of their affiliated organizations, or those of the publisher, the editors and the reviewers. Any product that may be evaluated in this article, or claim that may be made by its manufacturer, is not guaranteed or endorsed by the publisher.
