# Supplementary material for: Antithrombotic Therapy With Ticagrelor in Atrial Fibrillation Subjects After Percutaneous Coronary Intervention
Source: Front Cardiovasc Med. 2021 Oct 12;8:745549. doi: 10.3389/fcvm.2021.745549 (PMC8545975; doi:10.3389/fcvm.2021.745549)
Supplement: Supplementary file 1 [file Data_Sheet_1.docx]

**Supplementary Figure 1.**

TT group

2014

2015、

2016

2017

2018

DT group

Number of patients

**Supplementary Figure 1.** Randomized enrollment progress in each year and patient distribution in the two groups are shown.

**Supplementary Table 1.**


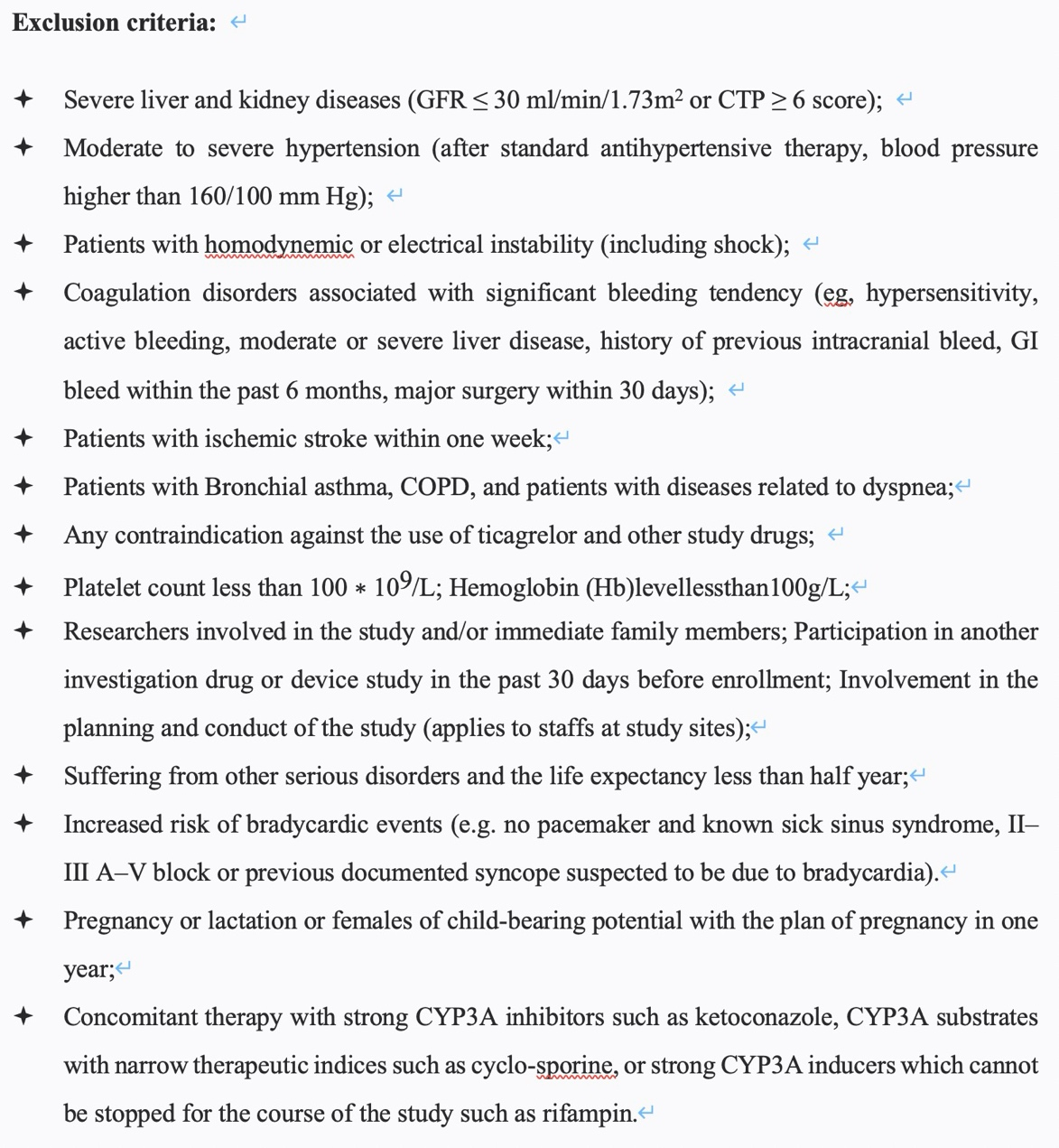


**Supplementary Table 1.** Exclusion Criteria of the Trial. COPD, chronic obstructive pulmonary disease; CTP, Child-Turcotte-Pugh score; GFR, glomerular filtration rate.

**Supplementary Table 2.**

| Outcome | Dual Therapy Group (n = 121) | Triple therapy Group  (n = 126) | Hazard Ratio (95% CI) | *P* value |
| --- | --- | --- | --- | --- |
| Bleeding |  |  |  |  |
| Total bleeding | 48 (39.67%) | 46 (36.51%) | 0.889 (0.593-1.332) | 0.5684 |
| Major bleeding | 6 (4.96%) | 2 (1.59%) | 0.313 (0.063-1.552) | 0.1550 |

**Supplementary Table 2.** Primary and Secondary Outcomes of the 2 Groups in PPS (N = 247). HR=hazard ratio. Data are expressed as n (%).

**Supplementary Table 3.**

| Outcome | Dual Therapy Group (n = 121) | Triple therapy Group (n = 126) | Hazard Ratio (95% CI) | *P* value |
| --- | --- | --- | --- | --- |
| Events | 22 (18.18%) | 20 (15.87%) | 0.844 (0.461-1.547) | 0.5836 |

**Supplementary Table 3.** Cardiovascular Events of 2 groups of patients in PPS (N = 247). HR=hazard ratio. Data are expressed as n (%).
